# Radiological patterns and pulmonary function values of lung involvement in primary Sjögren’s syndrome: A pilot analysis

**DOI:** 10.3389/fmed.2022.998028

**Published:** 2022-10-28

**Authors:** Ilaria Mormile, Mauro Mormile, Francesca Wanda Rossi, Michela Williams, Tullio Valente, Claudio Candia, Francescopaolo Granata, Roberto Rega, Martina Orlandi, Marco Matucci-Cerinic, Antonio Molino, Amato de Paulis

**Affiliations:** ^1^Department of Translational Medical Sciences, University of Naples Federico II, Naples, Italy; ^2^Department of Clinical Medicine and Surgery, University of Naples Federico II, Naples, Italy; ^3^Center for Basic and Clinical Immunology Research (CISI), WAO Center of Excellence, University of Naples Federico II, Naples, Italy; ^4^Department of Radiology, Monaldi Hospital, Azienda Ospedaliera dei Colli, Naples, Italy; ^5^Respiratory Division, Department of Respiratory Medicine, University of Naples Federico II, Naples, Italy; ^6^Department of Respiratory Medicine, AORN dei Colli, Naples, Italy; ^7^Division of Rheumatology AOUC, Department of Experimental and Clinical Medicine, University of Florence, Florence, Italy; ^8^Unit of Immunology, Rheumatology, Allergy, and Rare Diseases, IRCCS San Raffaele Hospital, Milan, Italy

**Keywords:** Sjögren’s syndrome, interstitial lung disease, tracheobronchial alterations, emphysema, primary Sjögren syndrome, bronchiectasis

## Abstract

**Background:**

Lung involvement in primary Sjögren’s syndrome (pSS) may vary from 9 to 90%. Interstitial lung disease and tracheobronchial alterations are the most typical findings. The evidence of primarily emphysematous changes at computed tomography of the chest of pSS patients has occasionally been described but poorly characterized. This study aims to assess pulmonary involvement and the impact on respiratory function in a cohort of pSS patients.

**Materials and methods:**

A total of 22 consecutive patients diagnosed with pSS underwent pulmonary function tests to investigate the presence of ventilatory impairment and evaluate the exchanges of alveolar gases. All patients underwent a chest high-resolution computed tomography (HRTC).

**Results:**

Dynamic volumes were within the normal range in 21 patients (95.4%). A reduction in the diffusing capacity of the lung for carbon monoxide (DLCO) was observed in 18 patients (81.8%). Ten (45.5%) patients showed a mild degree deficit, while 8 patients (36%) showed a moderate degree deficit. Analysis of DLCO revealed a significant difference between pSS patients and controls [*t*(30.98) = −10.77; *p* < 0.001], showing a higher DLCO value for the healthy controls (mean ± SE; 101.27 ± 6.08) compared to pSS patients (mean ± SE; 65.95 ± 12.78). Emphysema was found in 21 (94.5%) patients and was the most widespread pulmonary injury. Tracheal thickness was reduced in 15 (67%) patients. Micronodules were observed in 10 (45%) patients in all the pulmonary fields. Bronchial wall thickening and bronchiectasis were observed in 8 (36%) patients, mainly in the lower lobes. Ground glass was found in 5 (22.5%) patients in lower and higher lobes. Cysts were observed in two patients (9%).

**Conclusion:**

The reduction of the DLCO could be related to early emphysematous alterations in the absence of spirometric alterations and relevant respiratory symptoms. In conclusion, emphysema might be seen as an early pulmonary involvement mark in patients suffering from pSS.

## Introduction

Sjögren’s syndrome is characterized by lymphocytic infiltration of the exocrine glands resulting in damage and impaired function, mainly involving salivary and lacrimal glands, asthenia, joint pain, and an increased risk of lymphoid malignancies (1). It is significantly more frequent in women than men; the sex difference ranges from 9:1 to 19:1 (2). Sjögren’s syndrome is classified as primary Sjögren’s syndrome (pSS) and secondary Sjögren’s syndrome (sSS) (1). pSS occurs alone, while sSS presents as part of other autoimmune conditions, especially systemic lupus erythematosus (15–36%), rheumatoid arthritis (20–32%), limited and progressive systemic sclerosis (11–24%), and less frequently, multiple sclerosis, autoimmune hepatitis, and thyroiditis (3–5). Laboratory testing may reveal high erythrocyte sedimentation rate (ESR), positive antinuclear antibody (ANA) in up to 83% of patients with pSS (6), anti-Ro/SSA and/or anti-La/SSB antibodies, respectively, detected in 40–75 and 23–52% of pSS patients (7), and low C4 levels (4).

Sjögren’s syndrome is a systemic disease affecting several organs and systems, including the cardiovascular, respiratory, gastrointestinal, musculoskeletal, central and peripheral nervous systems, and kidneys (1).

Respiratory symptoms due to the upper respiratory tract and large and small airway disease (e.g., cough and dyspnea) occur in 9–20% of patients (8). However, subclinical lung disease, including small airway disease and airway inflammation, is even more common (9). It has been estimated that lung involvement in Sjögren’s syndrome may vary from 9 to 90% (10–15), affecting airways, lung parenchyma, lymphoid structures, pleura, and pulmonary vascular components (16). Among these clinical manifestations, the most typical findings are interstitial lung disease (ILD) and tracheobronchial alterations (16). The evidence of primarily emphysematous changes at computed tomography (CT) of the chest of Sjögren’s syndrome patients has occasionally been described (10, 17, 18), but poorly characterized. This study aims to assess the pulmonary involvement and the impact on respiratory function in a cohort of 22 pSS patients.

## Materials and methods

### Patients

Twenty-two consecutive pSS patients followed at the Division of Internal Medicine and Clinical Immunology of the University of Naples Federico II, Naples, Italy, were enrolled in this cross-sectional single-center cohort study. The diagnosis was performed according to the American College of Rheumatology/European League Against Rheumatism (ACR-EULAR) pSS classification criteria (19). We included patients aged > 18 years, with available data on sex, date of birth, age of onset, pSS diagnosis age, smoking habit, and signature of the written informed consent. Patients with other systemic autoimmune rheumatic diseases, active hepatitis C, IgG4-dependent disease, or sarcoidosis were excluded for ruling out secondary causes of Sjögren’s syndrome (3). We excluded patients with known pulmonary and extrapulmonary conditions (e.g., neuromuscular diseases, pulmonary hypertension, congestive heart failure) to minimize their influence on the pulmonary function tests. We also excluded patients with environmental or occupational exposure which might lead to lung disease (e.g., asbestos, radon, free crystalline silica exposure) to rule out confounding factors for emphysema.

We enrolled 11 age-, sex, and tobacco use matched healthy controls chosen among non-respiratory patients who had undergone pulmonary function tests and a chest HRCT for screening purposes or before a non-thoracic surgery. Both patients and healthy controls lived in the same metropolitan area of Naples.

Non-smokers were classified as zero pack-year. Low, medium, and high smoking exposure were classified as one-five, five-ten, and greater than ten pack-years, respectively.

Clinical and laboratory data were collected from November 2018 to February 2020. Demographic and clinical data were collected from patient medical charts and diaries. Clinical items in the analysis were age, gender, smoking, and clinical features, including the presence/absence of respiratory symptoms (cough, dyspnea, phlegm, hemoptysis, and chest pain). At the time of evaluation, the EULAR Sjögren’s Syndrome Patient Reported Index (ESSPRI) and the EULAR Sjögren’s syndrome disease activity index (ESSDAI) were administered to all patients (20–22).

The study was conducted in accordance with the Declaration of Helsinki and approved by the Ethics Committee of the University of Naples Federico II. Prior to study participation, patients signed an informed consent form.

### Laboratory studies

Laboratory data included ESR, C-reactive protein (CRP), serum protein electrophoresis, complement levels, ANA, extractable nuclear antigen (ENA: Ro/SSA, La/SSB, Sm, Scl70, Jo1, histone, centromere), and rheumatoid factor (RF).

### Pulmonary function tests in Sjögren’s syndrome

Pulmonary function tests [i.e., spirometry with Forced Vital Capacity (FVC), diffusing capacity of the lung for carbon monoxide (DLCO), six-min walking test (6MWT), and arterial blood gas analysis (ABG)] were performed at the Respiratory Division, Department of Respiratory Medicine, University of Naples Federico II, Naples, Italy, on both patients and healthy controls. All patients underwent pulmonary function tests to identify an obstructive, restrictive or mixed syndrome and to assess any impairment in the exchanges of alveolar gases and reduced tolerance to physical exercise (23, 24). Respiratory function tests were reported according to the latest ERS/ATS guidelines (25–27). In detail, as regards spirometric outcomes, the obstructive respiratory pattern was diagnosed when Forced Vital Capacity (FVC) was in the range of normality and the ratio FEV1/FVC was lower to 70% after administration of salbutamol 400 mcg; the restrictive respiratory pattern was diagnosed when both Forced Expiratory Volume in 1st second (FEV1) and FVC were below the Lower Limit of Normality, and the ratio FEV1/FVC was >80%; the mixed respiratory pattern was diagnosed when both FEV1 and FVC were impaired, but the ratio FEV1/FVC was <80 and >70% (25, 26). The spirometric test was performed with Master Screen Body^®^ Jaeger–Carefusion spirometer (22745 Savi Ranch Parkway, Yorba Linda, CA, USA). It was repeated until three acceptable spirograms were obtained (i.e., free of artifacts, satisfactory exhalation, a 6-s plateau at the end of the forced expiratory phase, and appropriate patient compliance). Peak expiratory flow (PEF), FVC, FEV1, the FEV1/FVC ratio, and the residual volume (RV) were also measured to evaluate pulmonary function. All the results were compared to reference values for adult patients matched for age, sex, and height.

Diffusing capacity of the lung for carbon monoxide was measured *via* single-breath technique and was performed with Master Screen Body^®^ Jaeger–Carefusion spirometer (22745 Savi Ranch Parkway, Yorba Linda, CA, USA).

According to ATS guidelines, the 6MWT was carried on a straight indoor line (28). All participants scored their level of dyspnea and overall fatigue based on a modified Borg scale using values from 0 (no dyspnea) to 10 (maximal exhaustion) (29). Blood pressure, pulse rate, oxygen saturation, and a physician evaluation were assessed at the beginning and the end of the test. In addition, all patients underwent an ABG analysis to assess their oxygenation status and acid-base balance.

### High-resolution computed tomography

All patients underwent chest high-resolution computed tomography (HRTC) with a 16-slice multi-detector CT scanner (MDCT 16 Brilliance Philips, Eindhoven, The Netherlands) and a 64-slice multi-detector CT scanner (MDCT 64, General Electric Medical System, Milwaukee, WI, USA), with patients in the supine position, at full inspiration. Scanning parameters were 120 kV and 240 mAs by applying the smallest field of view according to the patient body habitus. Matrix size was 512 × 512 pixels; images were reconstructed with a 1/1.25 mm slice thickness using bone filters. The whole chest volume was processed and stored on a picture archiving and communication system for post-processing evaluation (DICOM images). Lung parenchyma was independently analyzed by a 20 years of experience radiologist blinded to the clinical symptoms, and functional results, with a window width of 1.600 Hounsfield Units (HU) and level −600 HU. No intravenous contrast material was administered.

Emphysema index (in pSS-cohort and the control group) was always defined as voxels ≤−950 HU on inspiratory scans and expressed as percentage of TLCCT (total lung capacity measured by CT) (30). Emphysema was also graded with a 5-point scale-based on the percentage of lung involved: 0 = no emphysema, 1 = up to 25% of lung parenchyma involved, 2 = 26–50, 3 = 51–75, and 4 = 76–100% of lung parenchyma involved (31).

The thin-section HRCT findings were recorded according to the Fleischner society 2008 glossary of terms for thoracic imaging (32): (i) bronchial wall thickening (if the lobar bronchial wall was >3.0 mm in thickness on lung window images; segmental and subsegmental bronchi when their walls were >1.5 mm in thickness), bronchiectasis; (ii) tracheal wall measurement; (iii) small parenchymal nodules (short-axis diameter < 10 mm), large nodules (10–30 mm), and mass (more than 30 mm); centrilobular/perilymphatic/random nodules, tree-in-bud pattern; (iv) reticular pattern, including thickened interlobular septa and intralobular reticular lines; (v) linear opacities; (vi) architectural distortion, traction bronchiectasis, and bronchiolectasis, honeycombing; (vii) emphysema; (viii) increased lung attenuation, including ground-glass attenuation, air-space consolidation, and atelectasis; (ix) decreased lung attenuation with mosaic perfusion, air trapping; (x) thin-walled lung cysts; (xi) artery enlargement; (xii) pleural abnormalities, hilar and mediastinal lymphadenopathy and/or masses. All 12 findings were assessed regarding their presence, extension, and distribution (upper lobe, right middle lobe, lingula, and lower lobe; central, peripheral, diffuse, or random).

**FIGURE 1:**
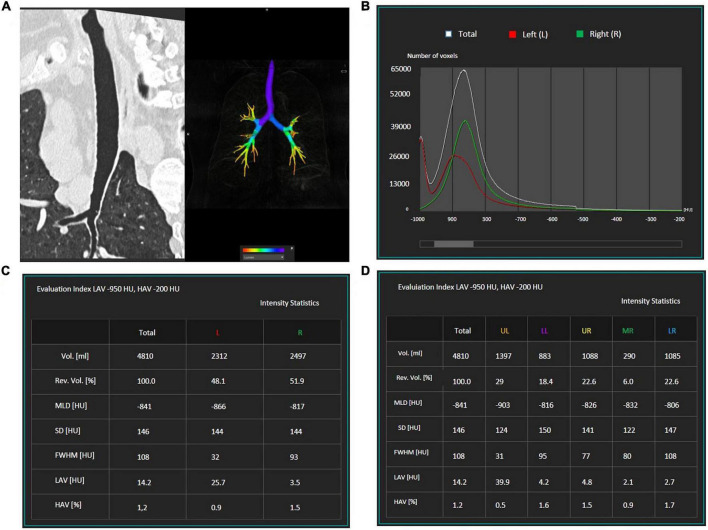
Example of the semi-automatic software which was used for emphysema quantification. First, the airways **(A)** lungs, and lobes are segmented. A graphic **(B)** displays the number of voxels with a specific density: emphysematous areas are characterized by a density lower than –950 HU. The software quantifies lung volumes and recognizes the total volume of emphysematous areas [lower attenuation areas (LAV)] for each lung **(C)**. The software can be used to calculate the volume of emphysematous areas within each lung lobe **(D)**.

For automatic quantification of the extent of emphysema, HRCT images were loaded at a commercially available workstation (IntelliSpace version 8, COPD tool, Philips Healthcare, Best, The Netherlands), which calculates on density histograms the percentage of lung voxels with X-ray attenuation values lower than −950 Hounsfield units (HU) (percentage low attenuation areas,% LAA) separately on the right and left lobe/lung or on the whole lungs volume after 3D airways, lungs, and lobes segmentation (Figure 1). The −950 HU cut-off has been previously shown to be appropriate for this CT acquisition density-based technique (33, 34). The quality of each 3D segmentation was visually checked by the operator before the inclusion of the densitometric data in the analysis. The mean time for 3D segmentation of the whole lung was 15 s (range 7–20 s).

For tracheal wall thickness, evaluation images can be quickly reconstructed with thin sections in the off-axis axial plane and thin-slab MIP post-processing, and 3D imaging techniques (including external and internal volume rendering).

### Data analysis

Data were summarized by descriptive analysis. Means and SD were calculated for continuous variables, while absolute values and frequency (percentage) were calculated for categorical variables.

Values were presented as frequency (number and percentage) and mean ± standard error of the mean (SEM). Analysis of the dependent variable DLCO was performed with an unpaired *t*-test comparing pSS patients and healthy controls. Analysis of the dependent variable “percentage of emphysema” was performed with the Mann–Whitney test comparing patients and controls. To test the predictive value of ESR, gamma fraction of serum protein electrophoresis, and ESSPRI on DLCO outcome, we used linear regression analysis. The assumption of normality distribution of data residuals was assessed with Shapiro–Wilks’ test. The assumption of homoscedasticity was performed with Levene test. The level of significance was set at α = 0.05.

## Results

### Demographic data and clinical evaluation

The main clinical features of the patients are summarized in Table 1.

**TABLE 1:** Clinical features in our cohort of patients with primary Sjögren’s syndrome (pSS) (*N* = 22).

Patients’ features	Number of patients (%)
Female gender (*n*, %)	22 (100%)
Caucasian ethnicity (*n*, %)	22 (100%)
Age, years (mean ± SD, range)	57.7 ± 11.8 (36–80)
Smokers (*n*, %)	3 (13.6%)
Non-smokers (*n*, %)	13 (59%)
Former smokers (*n*, %)	6 (27.2%)
Low pack/years (<5) (*n*, %)	2 (9%)
Medium pack/years (5–10) (*n*, %)	1 (4.5%)
High pack/years (>10) (*n*, %)	4 (18.1%)
Unknown (*n*, %)	2 (9%)
Disease activity (ESSPRI) mean ± SD, range	7.32 ± 1.79 (4.6–10)
Disease activity (ESSDAI) mean ± SD, range	4.4 ± 6.4 (2–28)
**Treatment for pSS**	
Prednisone < 20 mg/day (*n*, %)	11 (50%)
Prednisone > 20 mg/day (*n*, %)	1 (4.5%)
Hydroxychloroquine (*n*, %)	11 (50%)
Methotrexate (*n*, %)	3 (13.6%)

ESSPRI, EULAR Sjögren’s Syndrome Patient Reported Index; ESSDAI, EULAR Sjögren’s syndrome disease activity index.

All 22 pSS patients enrolled were White Caucasian females (100%): the mean age at diagnosis was 57.7 ± 11.8 years (36–80), and the mean disease duration was 14.6 ± 8.2 years. The patients were followed for an average time of 3 ± 1.1 months (2–6). Moreover, 13 (59%) patients never smoked, 3 (13.6%) were current smokers, and 6 (27.2%) were former smokers. Patients’ pack-year exposure is displayed in Table 1. In our cohort, only 4 (18.1%) patients present a high pack/years (>10) exposure. For two patients, smoking was reported but pack-year information was unavailable.

Over half of the cohort (13 out of 22; 59%) presented with at least one of the respiratory symptoms investigated. In detail, 11 patients (50%) reported dyspnea, which was the most common respiratory symptom in our cohort, six patients (27.2%) cough, two patients (9%) phlegm, and two patients (9%) chest pain. No patient presented with hemoptysis. The average ESSPRI was 7.32 ± 1.79 (range 4.6–10) and the average ESSDAI 4.4 ± 6.4 (range 2–28). At the time of the assessment, 10 patients (45.4%) were treated with medium-low doses of prednisone (≤10 mg/day), one patient (4.5%) with methylprednisolone (8 mg/day), and 11 (50%) with hydroxychloroquine (200–400 mg/day).

Previous and current treatment with methotrexate was investigated for its potential lung toxicity (35).

### Laboratory tests

In 16 patients, ESR was increased (72.7%) (mean value 36.2 ± 23.9 mm/Ih–range 2–106; reference range < 15 mm/Ih), but CRP was negative in 17 patients (77.2%) and mildly elevated in other five patients (22.7%) (mean value 0.5 ± 23.9 mg/L–range 0.03–2.3, reference range < 0.3). C3 and C4 levels were decreased in 2 (9%) patients (C3 mean value 1.34 ± 0.19 g/L, reference range 0.9–1.8; C4 mean value 0.2 ± 0.08 g/L, reference range 0.1–0.4). ANA was positive in 15 (68.1%) patients (range 1/160–1/640). At baseline SSA and SSB antibodies were positive in 12 (54.5%) and 9 (40.9%) patients. Sm, Scl70, and Jo1 were negative, while RNP, histone, and centromere were each one positive in three patients, respectively. RF was positive in 5 (22.7%) patients (mean value 42.7 ± 148.8 UI/mL–range 0–687, reference range < 15 UI/mL). At serum protein electrophoresis, gamma fraction (%) was increased in 10 (45.4%) patients (mean value 18.7 ± 0.6%; range 12.2–40.7 or 1.49 ± 0.6 g/dl, range 0.8–4.11, reference range 11.1–18.8%, or 10.3–18.2 g/dl).

In all patients, ABG analysis was within range reference showing PH mean value 7.4 ± 0.02 (range 7.37–7.49, reference range 7.35–7.45), PaO_2_ mean value 89.9 ± 10.4 mmHg (range 76–115, reference range 75–100 mmHg), PaCO_2_ mean value 36.5 ± 4.3 mmHg (range 29–43, reference range 35–45 mmHg).

### Pulmonary function testing

**FIGURE 2:**
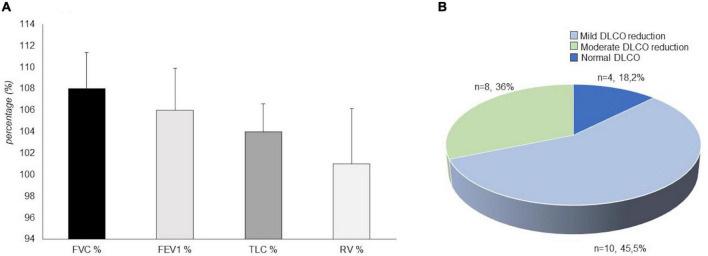
Mean value of spirometry parameters in our patients with primary Sjögren’s syndrome (pSS) (*N* = 22). FVC%, Vital capacity forced expressed in a percentage value of the theoretical; FEV1%, Forced expiratory volume in the first second expressed in a percentage value of the theoretical; TLC%, total lung capacity expressed in a percentage value of the theoretical; RV%, Residual volume expressed in a percentage value of the theoretical **(A)**. Diffusing capacity of the lung for carbon monoxide (DLCO) outcome in our patients with primary Sjögren’s syndrome (*N* = 22). In total, four patients (18.2%) showed a normal pattern, 10 patients (45.5%) showed a mild degree deficit, eight patients (36%) showed a moderate degree deficit **(B)**.

**FIGURE 3:**
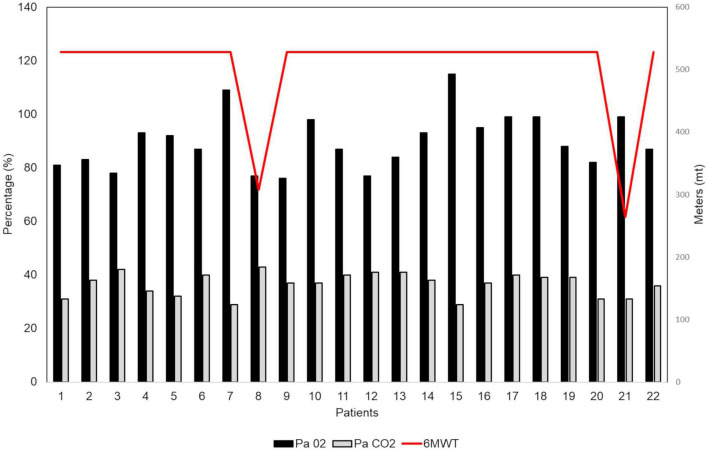
Six-min walking test (6MWT) in our patients with primary Sjögren’s syndrome (pSS) (*N* = 22). At the 6MWT 20 patients (92%) have traveled more than 500 m and 2 patients (9%) have traveled less than 500 m (red line). All patients underwent an arterial blood gas (ABG) analysis for assessing their oxygenation status (PaO_2_ = black column; PaCO_2_ = gray column) and acid-base balance.

Dynamic volumes were within the normal range in 21 (95.4%) patients: FEV1 (%) had an average value of 106 ± 18.37; FVC had an average value of 108% ± 15.7; RV was on average 101 ± 24.3%. A patient (4.5%), who has never been a smoker, presented an obstructive defect with a 63% FEV1, 69% FVC and an increase in RV (160%) (Figure 2A). A DLCO reduction was observed in 18 patients (81.8%). The average value was 65.95 ± 12.78%. 10 (45.5%) of patients showed a mild degree deficit, while eight patients (36%) showed a moderate degree deficit (25) (Figure 2B). Analysis of DLCO revealed a significant difference between pSS patients and controls [*t*(30.98) = −10.77; *p* < 0.001], showing a higher DLCO value for the healthy controls (mean ± SE; 101.27 ± 6.08) compared to pSS patients (mean ± SE; 65.95 ± 12.78). At the 6MWT, 20 patients (92%) walked more than 500 m, and two (9%) traveled less than 500 m. Of the two patients, one has traveled 308 m in 6 min, in the absence of interruptions, reporting slight effort dyspnea attributed to excessive body weight, while the other patient has traveled 264 mt, performing different interruptions due to hip osteoarthritis (Figure 3) (25).

Regression analysis showed that ESR (*R* = 0.16; *p* = 0.48), gamma fraction of serum protein electrophoresis (*R* = 0.11; *p* = 0.61), and ESSPRI were not significant predictors of DLCO outcome (*R* = 0.19; *p* = 0.49).

### Radiological findings

**FIGURE 4:**
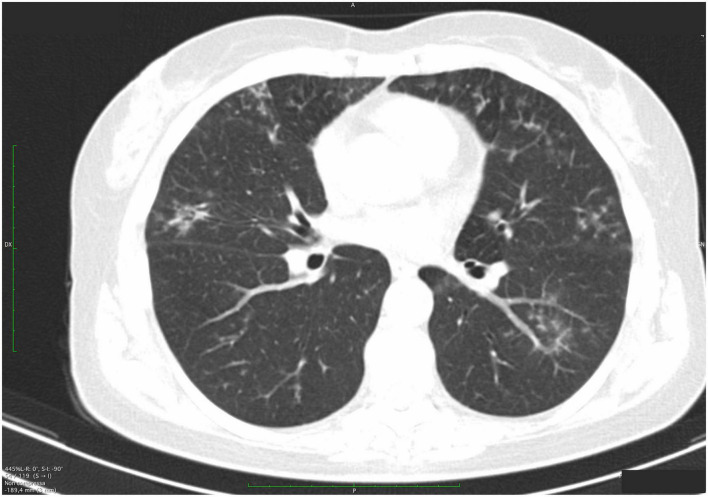
High-resolution computed tomography (HRCT) axial scans of a patient with primary Sjögren’s syndrome (pSS). The patient was a 54-year-old woman with a mild non-productive cough. Multiple semisolid and ill-defined margins centrilobular small nodules are observed, predominantly along the subpleural region, with subpleural interlobular septal thickening. In the right upper lobe, bronchiectasis and early cysts with adjacent peribronchiolar ground-glass thickening are found).

**FIGURE 5:**
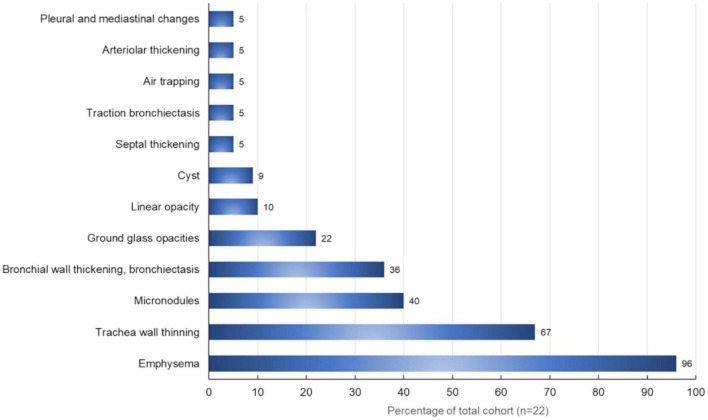
Radiological findings (%) in our patients with primary Sjögren’s syndrome (*N* = 22) evaluated through high-resolution computed tomography.

The different radiological aspects detected have been evaluated, considering their extension and distribution. Emphysema was present in 21 (95.4%) patients, resulting in the most widespread pulmonary condition, followed by the reduction (normal values > 0.15 cm) of the tracheal thickness (15 out of 22 patients, 67%). Micronodules (Figure 4) were present in all pulmonary fields in 10 (45%) patients. In our cohort, the frequency of the most common radiological findings is summarized in Figure 5.

On HRCT, emphysema percentage was observed in 21 (95.4%) patients. Moreover, a 3D reconstruction showed that emphysema was, on average, 20 ± 12%.

Analysis of emphysema percentage did not reveal a significant difference between the pSS patients and healthy controls [*U* = 106.50; *p* = 0.58].

The smokers (*n* = 3) and former smokers (*n* = 6) (*n* total = 9/22 = 41%), have shown more evidence of airways remodeling (*n* = 3/9), parenchymal micronodules (*n* = 3/9), patchy (*n* = 1/9) or gravitational GGO (*n* = 1/9). Finally, 6 out 9 patients (66%) showed a percentage of emphysema > 15%.

## Discussion

In several studies, the involvement of the respiratory system has been described in pSS with a prevalence ranging from 9 to 90% (10, 11). It may affect airways, lung parenchyma, lymphoid structures, pleural, and pulmonary vascular components (11). One of the most typical manifestations is tracheobronchial disease, characterized by diffuse lymphocytic infiltration of the airway (11). It may cause bronchial wall thickening with hyperresponsiveness, bronchiectasis, bronchiolitis, recurrent respiratory infections, centrilobular lung nodules, and branching nodular opacities (11). Similarly, some of the radiological findings in our pSS cohort included thickening of the alveolar septs as well as diffuse bronchiectasis. Most commonly, ILD (10) is a fibrotic variant of the “non-specific” interstitial pneumonia (NSIP), while other histopathological patterns [i.e., usual interstitial pneumonia (UIP), organizing pneumonia, lymphocytic interstitial pneumonia (LIP)] are rare (10).

The prevalence of combined emphysema and fibrosis has been previously reported by Cottin et al. (17) in a multicenter retrospective study analyzing 34 patients (88% currently or former smokers) with several diagnoses of connective diseases (among them one Sjögren’s syndrome patient). In a retrospective study, Lohrmann et al. (10) estimated a 28% of a radiological emphysematous pattern in non-smoker pSS patients: the authors underlined the relevant HRCT contribution to the characterization of the wide variety of lung abnormalities in pSS. Indeed, HRTC may detect pulmonary changes before the onset of respiratory symptoms and delineate parenchymal abnormalities in limited lung areas, while a measurable impairment of lung function requires a larger lung segment (10). However, spirometry is a useful tool for evaluating obstructive airway disorders, restriction, or mixed diseases in pSS patients (23). Indeed, although we did not observe any functional alterations most of our pSS patients, obstructive, restrictive, and mixed ventilator impairment may occur in pSS (11, 36–38). Airway disease in Sjögren’s syndrome is caused by the destruction of exocrine glands or cell infiltration: this process may affect the trachea, central and peripheral bronchi, and bronchioles (10). The estimated frequency of distal airway disease in Sjögren’s syndrome varies from 22 to 46% (10). Obstructive ventilator syndrome is generally considered rare and seems to be related to disease severity (10, 11). Previously, Segal et al. (39) reported that airway obstruction was the most frequent alteration observed in 34% of 20 non-smoker Sjögren’s syndrome patients. More recently, Nilsson et al. (11) observed that the overall prevalence of obstructive syndrome in pSS patients was 41%, while the prevalence among never-smoking pSS patients was 30%. In pSS, the authors observed that the vital capacity (VC), FEV1, and FEV1/FVC ratio significantly decreased with the DLCO, while RV and the RV/total lung capacity ratio were significantly increased (11). A moderate correlation was observed between the disease activity and the pulmonary function test. These results, taken together, suggest that pSS itself may be involved in the development of chronic obstructive lung disease (11).

An 81.8% DLCO decrease was detected with a normal FVC. Normal spirometry and lung volumes with low DLCO can characterize pulmonary vascular diseases, early ILD, or emphysema (40). In detecting some pathological alterations, DLCO seems to be more sensible than spirometry. In addition, connective tissue disease-related ILD is often asymptomatic, with symptoms occurring at a late disease stage (41, 42). Therefore, lung screening in pSS with pulmonary function tests can allow an early diagnosis and prompt therapy.

Although a small sample was examined, this study offers important insights. In pSS, the evidence of emphysema at HRTC with a concomitant DLCO decrease and normal FVC could be an expression of early lung involvement. The aim of our study was focused on evaluating underestimated pulmonary manifestations. It is likely that in our cohort, the higher percentage of patients affected by emphysema in comparison to what was observed by other authors is related to the specific analysis performed in the context of the HRTC, which takes advantage of a commercially available workstation for automatic quantification of the extent of emphysema (10, 43). Our findings could be a starting point to plan a multi-center trial for gaining insights into pulmonary manifestation in pSS patients beyond ILD. The pathogenesis of emphysema in pSS is still unclear. It might be hypothesized that alveolar septa are invaded by lymphoid cells, thus leading to interstitial thickening or modification of the alveolar structure up to cysts and emphysema. In our cohort, most patients (21/22) developed an emphysema, while the structure modifications have been shown in two patients only. One of these patients had reduced functional tests with FEV1 63%/Th, FVC 69%/Th, and a significant DLCO decline (52%/Th).

**FIGURE 6:**
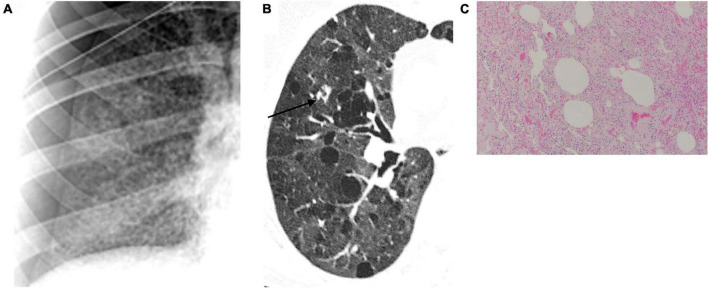
Lung cystogenesis. Usually, the “holes” appear black on chest X-ray **(A)** and high-resolution computed tomography **(B)** and white on pathological specimens **(C)**. Courtesy of Dr. Luigi Panico, Azienda Ospedaliera dei colli, Naples, Italy.

In our pSS cohort, the radiological findings strongly suggest that the formation of cysts is one of the pulmonary manifestations of the disease. A multidisciplinary approach should be used to diagnose cystic lung disease correctly, considering the patient’s clinical history, physical examination findings, and radiological appearance. On chest X-ray and chest HRCT scans, a lung cyst appears as a usually gas-filled round or irregularly shaped parenchymal lucency/low-attenuating area (“hole”) with (a well-defined interface) a thin (<2–4 mm) wall thickness, variable diameter, enclosed by an epithelial or fibrous wall, circumscribed by normal lung (32, 44). The cysts occasionally may contain fluid or solid material (45). Diseases characterized by true cysts are relatively uncommon and are usually distinguishable from their mimicking cyst-like entities (e.g., bullae, honeycombing, cavitated nodules, and bronchiectasis) that are not included in this manuscript (46). Pneumatoceles are thin-walled, gas-filled spaces associated with acute infections and after trauma and are considered a subcategory of usually transient cysts (32). Unlike other solid organs, the lungs do not develop so-called simple cysts. Usually, the “holes” appear black on chest X-ray and HRCT and white on pathological specimens (Figure 6). The pathogenesis of cyst formation in the lung is unclear. The main mechanisms may include: (i) check-valve obstruction by peribronchiolar infiltration/interstitial fibrosis with distal overinflation, (ii) ischemia, (iii) remodeling induced by MMPs and other matrix-degrading enzymes, and parenchymal (suppurative, caseous, and tumoral) necrosis (44, 46). Many cystic lung diseases have histopathological and imaging overlapping features and can be classified by the mechanism of cyst formation (cystogenesis) in more than one category. On the other hand, more than one mechanism may contribute to disease evolution. However, one of the most important factors leading to cyst formation is the bronchiolar and peribronchiolar infiltration upstream of the site of cyst formation, creating a ball valve effect. These check valves prevent air egress on exhalation while allowing air to enter on inspiration with subsequent distal over-inflation. Therefore, the coexistence of interstitial abnormalities in a small airway disease may be required for cyst formation. However, factors driving the evolution of pSS toward an emphysematous pattern rather than an interstitial one are still unclear, and future research should focus on this point.

Our study is subject to some limitations, including the single center design and the small sample size. However, despite the limited sample analyzed, this study may offer some interesting evidence, such as that DLCO decline might be due to emphysema rather than to ILD. In conclusion, emphysema might be a specific underestimated marker of progression of lung involvement in pSS patients.

## Data availability statement

The data underlying this article cannot be shared publicly due to the privacy of individuals that participated in the study. The data will be shared on reasonable request to the corresponding author FR.

## Ethics statement

The studies involving human participants were reviewed and approved by Ethics Committee of the University of Naples Federico II. The patients/participants provided their written informed consent to participate in this study.

## Author contributions

IM, TV, MM, AM, MO, MM-C, AP, and FR were responsible for study conception and design and were responsible for the analysis and interpretation of data. IM, TV, MW, CC, FG, and RR were responsible for the acquisition of data. All authors were involved in drafting the article or revising it critically for important intellectual content, also agreed to be accountable for all aspects of the work in ensuring that questions related to the accuracy or integrity of any part of the work are appropriately investigated and resolved, and approved the final version to be published.
